# Treatment decisions on Molar-Incisor Hypomineralization (MIH) by Norwegian dentists – a questionnaire study

**DOI:** 10.1186/s12903-016-0237-5

**Published:** 2016-07-04

**Authors:** Simen E. Kopperud, Cecilie Gravdahl Pedersen, Ivar Espelid

**Affiliations:** Department of Paediatric Dentistry and Behavioural Science, Faculty of Dentistry, University of Oslo, P.O. Box 1109, Blindern, NO-0317, Oslo, Norway; Nordic Institute of Dental Materials (NIOM), Oslo, Norway

**Keywords:** Molar incisor hypomineralization, MIH, Operative treatment, Composite resins, Dentistry

## Abstract

**Background:**

The study aimed to explore the variability between the treatment decisions dentists make for MIH-affected teeth.

**Methods:**

In 2009, a pre-coded questionnaire was sent electronically to all dentists employed by the Public Dental Service (PDS) in Norway (*n* = 1061). The questions were related to treatment of MIH-affected teeth, including three patient cases illustrated by photographs and written case descriptions.

**Results:**

Replies were obtained from 61.5 % of the respondents after two reminders. In the first case, showing a newly erupted first permanent molar with moderate hypomineralization and no disintegration of the surface enamel, the preferred treatment among the majority of the respondents (53.5 %) was application of fluoride varnish, while 19.6 % would seal the fissure with GIC material. In the second case, showing a severely damaged first permanent molar in a six year old child, more than half of the respondents (57.5 %) would place a conventional glass ionomer restoration and 10.5 % would use a stainless steel crown (SSC). In the third case, showing a severely damaged permanent first molar in a nine year old child, 43.8 % of the dentists would remove only the parts with soft, damaged enamel; while 35.2 % would remove more and 21.0 % would remove all affected enamel and leave the cavity margins in sound enamel.

**Conclusions:**

The survey shows that there is a wide disparity between clinicians’ views on how MIH affected teeth should be treated. In a severely affected first permanent molar, only a minority of dentists would remove as much tooth substance as needed to get the full benefit of the acid etch pattern in sound enamel.

## Background

The clinical expression of first permanent molars with Molar-Incisor Hypomineralization (MIH) represents a continuum of severity from hardly visible opacities to severe destruction of the enamel. In addition, affected teeth have a tendency to accumulate more severe defects over time, due to post-eruptive breakdown of hypomineralized enamel [1–4]. It is challenging for clinicians to make the best treatment decision in both a short and long term perspective. MIH-affected teeth are considered demanding to treat, due to difficulties in achieving adequate pain control, dental fear and behavioural management problems, determining the optimal preparation border and selecting an appropriate restorative material [5–7]. At present, there is no standard treatment which can be recommended for all MIH-affected teeth and the severity of the defects on MIH-affected teeth increases with patient’s age [8]. According to best clinical practice guidance and evaluation of relevant literature, resin composite is the recommended restorative material in the long run for fully erupted MIH-affected teeth [9].

In recent years, the term “minimally invasive dentistry” has been emphasised both in the education of new dentists and in continuing education programs for general practitioners in Norway, regarding treatment of caries. A mantra that “a dentist’s aim should be to avoid operative treatment wherever possible” [10] has been repeated for all Norwegian dentists through basic education and continuing education, and a considerable shift in the dentists’ criteria for operative treatment of dental caries in Norway has already been demonstrated [11]. However, is the minimally invasive approach the best alternative for MIH-affected teeth or should affected enamel be removed before restoration? Affected MIH enamel is less amenable to acid etching [12–15], which might weaken the retention of sealants [16, 17] and resin-based restorations [16]. According to studies by William and Mathu-Maju published in 2006, all affected or discoloured enamel should be removed to achieve the best bond [7, 18]. Nevertheless, results from a longitudinal study with 12 months follow up of 6 to 9 years old children indicate that complete removal of affected enamel is not justified [2], even though the value of such short-term clinical studies are limited. Reviews of studies on longevity of restorations would normally include only studies with at least 4–5 years observation time [19, 20].

The aim of our study was to explore the variability between dentists in treatment decisions of MIH-affected molars, using two specific patient cases with different grades of severity and one patient case where the dentists could choose how much enamel to remove. The research hypothesis was that Norwegian dentists follow the rules of minimally invasive dentistry strictly in cases where a more radical approach could be needed.

## Methods

Using the software Questback (Oslo, Norway), a pre-coded questionnaire was sent electronically in May 2009 to all dentists employed by the Public Dental Service (PDS) with an email address registered in the Norwegian Dental Association (NTF). Of the 1386 dentists employed by the PDS, 1245 email addresses were registered. The address-list was searched manually by the authors, and 184 dentists that we knew did not work clinically with a relevant patient population were excluded (e.g., dentists with specialities in other fields, dentists occupied with research, administrative work, etc.). Anonymity of the respondents was ensured by QuestBack. The study was approved by the Norwegian Social Science Data Services (NSD) (Project number 21434).

**Fig. 1:**
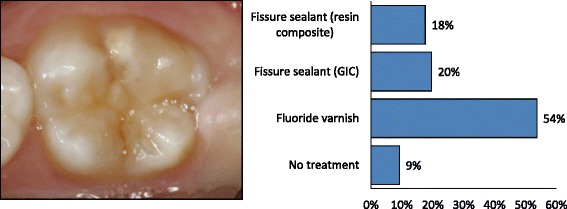
Which treatment would you provide for this newly erupted first molar with moderate hypomineralizations and no disintegration of the surface enamel? The patient is six years old, has good oral hygiene, normal occlusion and is cooperative

**Fig. 2:**
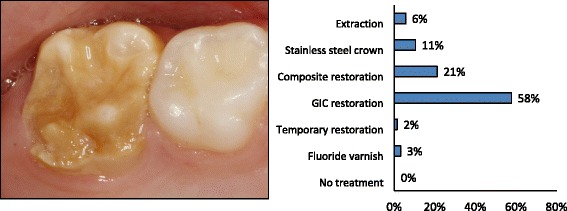
Which treatment would you provide for this newly erupted, severely damaged first permanent molar with post-eruptive breakdown? The tooth is sensitive to air. The patient is six years old, has good oral hygiene, normal occlusion and is cooperative

**Fig. 3:**
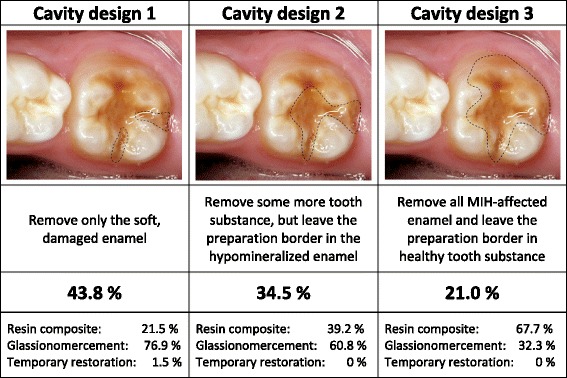
Where would you place the preparation margin if restoring this MIH-affected first permanent molar, and which restorative material would you use? The patient is nine years old, has good oral hygiene, normal occlusion and is cooperative

Information was collected on the respondent’s age, sex and demographic information. The questionnaire had two parts. The first part addressed the dentist’s clinical experience with paediatric dentistry, specifically around MIH-affected teeth, and their attitudes towards treatment of children. The dentist’s opinion on probable causes related to MIH was also recorded. In the second part of the questionnaire, the dentists were presented with three different patient cases with illustrative photographs and a written case description (Figs. 1, 2 and 3). In *Patient Case 1* the dentists were asked what treatment they would prefer on a newly erupted permanent first molar (Fig. 1) with moderate hypomineralizations and no disintegration of the surface enamel. The alternatives were: (1) No treatment, (2) Fluoride varnish, (3) Fissure sealant with glass ionomer based material, (4) Fissure sealant with resin composite based material. *Patient Case 2* showed a severely damaged first permanent molar with post-eruptive breakdown (Fig. 2) in a 6-year-old child with good cooperation, adequate oral hygiene and normal occlusion. The respondents were asked what treatment they preferred among the alternatives: (1) No treatment, (2) Fluoride varnish, (3) Temporary restoration with IRM, (4) Restoration in glass ionomer cement (GIC), (5) Restoration in resin composite, (6) Stainless Steel Crown (SSC), or (7) Extraction of the tooth. In the third case, a severely damaged first permanent molar was presented with three alternative cavity designs (Fig. 3): (1) Remove only the soft, damaged enamel, (2) Remove some more tooth substance, but leave the preparation border in the hypomineralized enamel, (3) Remove all MIH-affected enamel and leave the preparation border in healthy tooth substance. The patient was described being nine years old, cooperative, with adequate oral hygiene and normal occlusion.

The software (Questback) was configured to send automatically two reminders to all participants who did not reply within a certain time. Anonymity of the respondents was ensured by Questback. The study was approved by the Norwegian Social Science Data Services (NSD). Statistical analyses were performed with IBM SPSS Statistics version 20.0.0.1 (Statistical Package for the Social Sciences; SPSS, Chicago, IL, USA). Statistical evaluation was carried out by means of descriptive statistics with chi-square tests. A significance level of 5 % was used throughout.

## Results

Replies were received from 669 respondents after two reminders. Dentists 69 years of age and older (*n* = 36) and dentists not in active clinical practice (*n* = 27) were excluded. Thus, answers from 606 respondents were further processed in the final statistical analyses. A response rate of 61.5 % was calculated according to Standard Definitions of the American Association for Public Opinion Research [21]. The age of the included respondents varied from 29 to 68 years (mean 49, SD ±12), 64 % were female and 36 % male. Similar data for all PDS-employed dentists were extracted from Statistics Norway, Dental Health [22]. Our sample was found not to be significantly different from all dentists regarding sex (p = 0.07), but significantly fewer dentists in the lowest age group (<30 years) responded (0.01).

Fewer than half the dentists (44.5 %) reported that 30–60 % of their working day was spent on treating children (3–18 years), while 39 % reported using more than 60 % of their time on treatment of children. Of all dentists, 48.2 % found that children with MIH-affected teeth were more anxious compared with other children, while 43.7 % reported no difference and 1.3 % reported that they found children with MIH-affected teeth easier to treat; 5.6 % did not know. Regarding pain control, more than half the dentists found MIH-molars more difficult to anaesthetise, 35.5 % reported no difference and 7.7 % did not know. Only 12.5 % of the dentists had tried administering pain killing medications prior to treatment of MIH-teeth, while 39 % had experience with sedation of MIH-patients. About a fifth (21.6 %) of the respondents reported that they had used crowns (SSCs) on MIH-teeth, but during the last six months the average number of SSCs made was only 1.7 (SD ±1.4, range 0–8). Extraction of MIH-affected molars had never been performed by 32.3 % of the respondents. For the dentists who had extracted (67.7 %), a specialist in orthodontics had been consulted in most cases (88.7 %). Less than one-third of the respondents (27.8 %) had referred patients with MIH-affected teeth to other dentists or specialists in pedodontics, but more than half the dentists (59.6 %) claimed that they would have liked to refer MIH-patients had this been possible. A majority (82.2 %) of the dentists routinely recalled MIH-patients more often than other patients.

In the first patient case, showing a newly erupted first permanent molar with moderate hypomineralization and no disintegration of the surface enamel (Fig. 1), the preferred treatment among the majority of the respondents (53.5 %) was application of fluoride varnish, while 19.6 % would seal the fissure with GIC material. In the second patient case, showing a severely damaged first permanent molar in a 6-year-old child (Fig. 2), more than half of the respondents (57.5 %) would place a GIC restoration, 21.1 % suggested a resin composite restoration and 10.5 % preferred placing a SSC. In the third patient case, showing a severely damaged permanent first molar in a nine year old child (Fig. 3), 43.8 % of the dentists would remove only the parts with soft, damaged enamel (cavity design A), while 35.2 % would remove more (cavity design B) and 21.0 % would remove all affected enamel and leave the cavity margins in sound enamel (cavity design C). Of those who preferred cavity design A, 76.9 % chose GIC and 21.5 % resin composite as restorative material. Of those who preferred cavity design B, 60.8 % chose GIC and 39.2 % resin composite and of those who preferred cavity design C, 32.2 % chose GIC and 67.7 % resin composite.

## Discussion

The results show that there is a notable disparity between different clinicians’ treatment choices. On a newly erupted permanent first molar with moderate hypomineralization and no disintegration of the surface enamel, the majority of the respondents (51.2 %) preferred treatment with fluoride varnish. On a severely damaged first permanent molar in a 6-year-old child, more than half of the respondents (53.7 %), would place a conventional glass ionomer restoration. In a severely damaged permanent first molar in a nine year old child, only 21.0 % would remove all affected enamel and leave the cavity margins in sound enamel, which may reflect a preference for adhesive techniques. Of those who preferred cavity design C, 32.2 % chose glass ionomer cement and 67.7 % resin composite as restorative material.

The questionnaire was sent to all dentists employed by the Public Dental Service in Norway. The relatively high response rate (61.5 %) and the matching sex distribution are consistent with our sample being representative of all PDS-employed dentists in Norway, although significantly fewer of the younger dentists replied. All age cohorts in Norway up to the age of 20 years regularly attend the PDS and are enrolled in a recall program. In 2009, 95.6 % of all children and adolescents (aged 0–18) were under supervision and treatment by the PDS [22]. A limitation to the study is that the study was conducted seven years ago. Nevertheless, the findings are still likely to be valid. During the last seven years, the journal of the Norwegian Dental Association (NTF) has not published any article addressing the same topics as this survey covers, namely treatment decisions. Another limitation to the study is that the frequency of MIH-patients seen by respondents in our sample may vary, and consequently some dentists may have more recent experience with treatment of MIH. However, MIH is quite common in Norway. In an epidemiological study of 794 16-year old individuals in Norway 13.9 % were diagnosed with MIH [23].

*Patient Case 1* (Fig. 1) showed a newly erupted permanent first molar with moderate hypomineralizations and no disintegration of the surface enamel. The tooth was newly erupted and belonged to a 6 year old child indicating that there is a need for a decision on how to follow-up. In this case, 35 % of the dentists would choose to treat the tooth with fissure sealants (either glass ionomer or resin based materials). Fluoride varnish, which was chosen by more than 50 %, may reduce sensitivity [24] and possibly reduces the caries risk observed in MIH patients [25]. Almost 8 % of the dentists would do nothing. There are a few studies indicating that there is some potential for mineralisation of the porous MIH affected enamel or reduced hypersensitivity using CPP-ACFP (casein phosphopeptide - amorphous calcium fluoride phosphate) solution [24, 26], but so far more research is needed before this can be advocated on more general basis. In the authors’ opinion, a fissure sealant would be appropriate and probably a GIC based product would be preferable in the short run if moisture control could be difficult because an operculum covers the most distal part of the fissure. *Patient Case 2* (Fig. 2) showed a severely damaged FPM with post-eruptive breakdown in a 6 year old patient with a newly erupted, sensible first permanent molar. The case was supposed to reflect an unclear situation where dentists should consider a temporary, pain-relieving solution until the prognosis of the tooth becomes more certain or extract the tooth. In this patient case, almost 54 % of respondents chose to restore the affected areas with GIC. It may be questioned whether GIC has sufficient mechanical properties in stress bearing areas in MIH molars [6, 9], but as a temporary restoration, with fluoride release, dentists may consider GIC a “forgiving” material. The fluoride release may prevent both secondary caries and development of caries on the surface of the adjacent tooth. According to Lygidakis et al. [9], a conventional resin composite restoration or a SSC should be placed once breakdown has occurred depending of the severity of the hypomineralization. These are good alternatives in this case, since it was given that the child cooperated well. Only 19.1 % chose resin composite restoration and 9.8 % chose SSC in this patient case. If only two or three of the total five surfaces are affected, resin composite restorations show adequate long term performance and may be an alternative treatment to SSCs [27]. However, Mejàre et al. [6] found that conservative restorative treatment resulted in a need for additional retreatment in approximately half of the patients before reaching the age of eighteen. The median longevity of all kind of restorations in molars was 5.2 years. GIC had the lowest and resin composite the highest success rate. In *Patient Case 2* only 5.0 % of dentists chose extraction as the preferred treatment. Extraction of one or more molars has been claimed to be a good alternative in cases with heavily destroyed FPM [28]. Spontaneous space reduction and favourable development of the permanent dentition can be expected when extracting a severely damaged FPM before the eruption of the second permanent molar. Mejàre et al. [6] also found that extraction of molars with severe enamel defects gave good or acceptable results in most patients. Guidelines for such treatment are available [29]. The fact that Norwegian dentists did not use SSCs much could be due to little training in placing SSCs as students, partly due to low caries prevalence. Crombie et al. [5] found in their survey that SSCs were used significantly more by paediatric dentists (97 %) and postgraduate students in paediatric dentistry (100 %) compared with non-paediatric dentists (58 %). In our study, we did not distinguish between specialists in paediatric dentistry and non-paediatric dentists, because the number of practising specialists in Norway is very low. In the authors’ opinion, extraction might be considered as first choice. However, if the tooth should be kept for a shorter or longer period a SSC would be indicated. In *Patient Case 3* (Fig. 3) the dentists were asked to choose how much enamel they would remove based on three different situations. The patient was 9 year old and this illustrates a situation about three years after the molar eruption and that there is no acute symptoms and treatment needs. With respect to placement of preparation margin, only 21% would remove all affected enamel (cavity design C). In the authors’ opinion, this is the best treatment choice if a resin composite restoration is to be made. In the long term could an inlay or onlay be indicated if the composite needs revision, but this was not an option when the immediate treatment was to be decided. All other options than resin composite must be considered temporary solutions, since the etch pattern requires good retention and a tight seal [12–17]. Of the dentists who preferred cavity design C, 67.7 % would place a resin composite restoration and 32.2 % a GIC restoration. The latter may be considered a semi-permanent choice since the longevity of a GIC restoration in such a large cavity normally will be limited. Thus, in fact only 86 dentists (14.2 %) chose what the authors consider the best treatment alternative in this patient case. Most dentists chose cavity design A or B. Norwegian dentists are reluctant to remove tooth substance in general [11]. The concept of minimally invasive dentistry has been widely adopted by Norwegian dentists and it is likely that many dentists use this approach regularly even in cases where a more invasive approach could be beneficial, such as treatment of MIH. Another explanation could be that dentists find the patient group so challenging to treat that they limit tooth substance removal. Children with MIH-affected teeth show behavioural management problems (BMP) more often than other children [30]. It has been shown that MIH patients receive more restorations than do controls and they also get more caries [30, 31]. Even at the age of 18-years, MIH patients need more restorative dentistry [32]. One reason for the development of BMP could be difficulties in achieving adequate anaesthesia and the frequent dental treatments these children undergo. There is some evidence that the increased pain associated with MIH teeth has a biological explanation due to increased expression of a noxious heat receptor (TRPV1) in the pulp [33]. This emphasises the importance of using sedation with benzodiazepines, nitrous oxide or in severe cases general anaesthesia, when treating this patient group, both to be able to perform optimal treatment and to prevent the development of BMP. The hatched preparation border in cavity design C (Fig. 3) was supposed to illustrate that the preparation border was in healthy enamel. However, it is not easy to define the borderline between healthy and hypomineralized enamel. Fearne et al. [34] reported in one study that even visually normal enamel in these teeth was 5 % less mineralised than truly unaffected enamel. This five per cent deficit has been reported to result in dramatic reduction in the mechanical properties of hypomineralized teeth [35]. This could be one reason why these teeth frequently need retreatment after receiving restorations. In addition, the prismatic morphology in the porous enamel is altered, making bonding less effective [13].

## Conclusion

The survey shows that there is a wide disparity between clinicians’ views on how MIH affected teeth should be treated. In a severely affected first permanent molar, only a minority of dentists would remove as much tooth substance as needed to get the full benefit of the acid etch pattern in sound enamel. Thus, our research hypothesis was confirmed; Norwegian dentists follow the rules of minimally invasive dentistry too strictly in cases where a more radical approach may be needed. Continuing education of dentists in this field is still required. The great variation in treatment proposals among dentists indicate need for guidelines to minimize the treatment burden and secure high quality in treatment decisions and treatment. Treatment of severe MIH cases may be demanding and guiding or referral to specialists in paediatric dentistry might be beneficial. Children have rights to the enjoyment of the highest attainable standard of health [36] and the availability of paediatric dentists may limit this right.

## Abbreviations

BMP, behavioural management problems; FPM, first permanent molar; GIC, glass ionomer cement; MIH, molar-incisor hypomineralization; NSD, Norwegian Social Science Data Services; NTF, Norwegian Dental Association; PDS, Public Dental Service; SSC, stainless steel crown
